# Robust Pedestrian Classification Based on Hierarchical Kernel Sparse Representation

**DOI:** 10.3390/s16081296

**Published:** 2016-08-16

**Authors:** Rui Sun, Guanghai Zhang, Xiaoxing Yan, Jun Gao

**Affiliations:** 1 School of Computer and Information, Hefei University of Technology, Tunxi Road 193, Hefei 230009, China; zhangghai@yeah.net (G.Z.); gaojun@hfut.edu.cn (J.G.); 2 Academy of Optoelectronic Technology, Hefei University of Technology, Tunxi Road 193, Hefei 230009, China; yxxing@hfut.edu.cn

**Keywords:** pedestrian classification, CENTRIST, kernel method, sparse representation, pooling

## Abstract

Vision-based pedestrian detection has become an active topic in computer vision and autonomous vehicles. It aims at detecting pedestrians appearing ahead of the vehicle using a camera so that autonomous vehicles can assess the danger and take action. Due to varied illumination and appearance, complex background and occlusion pedestrian detection in outdoor environments is a difficult problem. In this paper, we propose a novel hierarchical feature extraction and weighted kernel sparse representation model for pedestrian classification. Initially, hierarchical feature extraction based on a CENTRIST descriptor is used to capture discriminative structures. A max pooling operation is used to enhance the invariance of varying appearance. Then, a kernel sparse representation model is proposed to fully exploit the discrimination information embedded in the hierarchical local features, and a Gaussian weight function as the measure to effectively handle the occlusion in pedestrian images. Extensive experiments are conducted on benchmark databases, including INRIA, Daimler, an artificially generated dataset and a real occluded dataset, demonstrating the more robust performance of the proposed method compared to state-of-the-art pedestrian classification methods.

## 1. Introduction

Pedestrian safety is an important problem for autonomous vehicles. A World Health Organization report describes road accidents as one of the significant causes of fatalities. About 10 million people become traffic casualties around the world each year, and two to three million of these people are seriously injured. The development of pedestrian protection systems (PPS) dedicated to reducing the number of fatalities and the severity of traffic accidents is an important and active research. PPS typically use forward vision sensors to detect pedestrians. Notwithstanding years of methodical and technical progress, e.g., see [1,2,3], pedestrian detection is still a difficult task from a machine-vision point of view. There is a wide range of pedestrian appearance arising from changing articulated pose, clothing, lighting and in case of a moving camera in a changing environment and partial occlusions pose additional problems. For different communities to benchmark and verify their pedestrian detection methods, many large-scale pedestrian data sets, including the Caltech [3], ETH [4], TUD-Brussels [5], Daimler [6], and INRIA [7] data sets, have been established and used as evaluation platforms.

Recently, some researchers and automobile manufacturers have tended to utilize advanced and expensive sensors such as infrared camera [8,9], radar [10], and laser scanners [11] in order to acquire much more information. The PPS of SAVE-U system contains a variety of sensors to achieve good system-level performance [12]. However, vision-based PPS is still a valuable strategy for onboard pedestrian detection due to the following advantages: (1) it is very cheap, which makes it a valuable solution for automobile manufacturers; (2) it has a longer detection range and good temperature characteristics; and (3) the key detection algorithms such as classification can be easily extended to other sensor systems.

A typical pedestrian detection algorithm can be divided into features extraction and classification. Marr claims that the primitives of visual information representation are simple components of forms and their local properties [13]. Therefore, local features-based methods are very promising in pedestrian detection. These features include Haar-like features [14], histogram of oriented gradient (HOG) [7], Gabor filter-based cortex features [15], covariance features [16], HOG-LBP features [17], edgelet features [18], shapelet features [19], CENTRIST [20], multiscale orientation features [21], etc. A recent survey [2] has shown that various HOG features are most effective for pedestrian detection.

While no single feature has been shown to outperform HOG, additional features can provide complementary information. Wojek and Schiele [22] show a combination of Haar-like features, shapelets, shape context and HOG features outperforms any individual feature. Walk et al. [23] extended this framework by additionally combining local color self-similarity and the motion features discussed in [22]. Likewise, Wu and Nevatia [24] automatically combined HOG, edgelet, and covariance features. Dollar et al. [25] proposed an extension of Haar-like features, which are computed over multiple channels of visual data, including LUV color channels, grayscale, gradient magnitude, and gradient magnitude quantized by orientation (implicitly computing gradient histograms), providing a simple and uniform framework for integrating multiple feature types. Unfortunately, multi-features improve detection accuracy but bring with it increased computational cost. Low computational requirements are of the essence for real-time onboard PPS.

In the classifiers, support vector machines (SVM) have become very popular in the domain of pedestrian classification, in both linear [7,26], and nonlinear variants [27]. Other popular classifiers include neural networks [28] and boosted classifiers [29]. Munder and Gavrila [30] studied the problem of pedestrian classification with different features and classifiers. They found that local receptive fields do a better job of representing pedestrians and that both SVM and adaboost classifiers outperformed the other tested classifiers. Xu et al. [31] proposed an efficient tree classifier ensemble-based method, which realize onboard detection in intelligent vehicles with a high detection speeds. Several approaches have attempted to break down the complexity of the problem into subparts. One way is to represent each body as an ensemble of components which are usually related to body parts. After detecting the individual body parts, detection results are fused using latent SVM [32], a Mixture-of-Experts framework [33], and the Restricted Boltzmann Machine Model [34].

Although these methods perform well under controlled conditions, they cannot handle effectively partially occluded, varying appearance and small-scale pedestrian images in a real-world scenario [2,35]. Recently an interesting classifier, namely sparse representation-based classification (SRC), was proposed by Wright et al. [36] for robust face recognition. Wright sparsely coded a testing image on the training set by L1-norm minimization, and then classified it to the class according to the least coding residual. By assuming that the outlier parts in the face image are sparse and by using an identity matrix to code the outliers, SRC has better classification performance than nearest neighbor (NN) [37], nearest subspace (NS) [38] and linear SVM [39] on face databases. However, SRC would lose its classification ability on data with the same direction distribution.

In this paper, we proposed a novel hierarchical features extraction and weighted kernel sparse representation (HFE − WKSR) model for pedestrian classification. First, we propose a hierarchical features extraction and max pooling (MP) operation to capture discriminative structures and enhance the invariance of varying appearance. Second, we propose a WKSR model, which not only uses kernel representation to fully exploit the discrimination information embedded in the hierarchical local features, but also adopts a Gaussian function as the measure to effectively handle the occlusion in query images. Compared with the previous classification methods, e.g., SVM with HOG features and SRC with holistic features, the proposed HFE − WKSR model shows much greater robustness with various pedestrian image variations (e.g., illumination, appearance and background) and partial occlusion, as demonstrated in our extensive experiments conducted on benchmark databases.

This paper is organized as follows. Section 2 briefly reviews some related work. Section 3 presents the proposed HFE − WKSR algorithm. Section 4 presents the experimental results. Section 5 summarizes this paper.

## 2. Related Work

### 2.1. CENTRIST Features

CENTRIST (CENsus TRansform hISTogram) is a histogram vector designed for establishing correspondence between local patches, firstly proposed for scene categorization [40]. Census transform (CT) compares the intensity value of a pixel with its eight neighboring pixels, as illustrated in Equation (1).

(1) $$\left[\begin{array}{ccc} 87 & 19 & 23\\ 23 & 27 & 15\\ 68 & 26 & 22 \end{array}\right]\Rightarrow \left[\begin{array}{ccc} 0 & 1 & 1\\ 1 & & 1\\ 0 & 1 & 1 \end{array}\right]\Rightarrow {\left(01111011\right)}_{2}\Rightarrow {\text{CT}=(123)}_{10}$$

CT compares the intensity value of a pixel with its 8-neighborhood. If the intensity value of the center pixel is bigger than (or equal to) one of its neighbors, a bit “1” is set in the corresponding location, otherwise a bit “0” is set. The eight bits stream generated from left to right, and top to bottom order, which is consequently converted to a base-10 number in [0, 255]. This is the CT value for the center pixel. After the pixel values are replaced by the CT values, the corresponding CT image is obtained. The CENTRIST descriptor is a histogram with 256 bins, which is a histogram of these CT values in an entire image or a rectangular region in an image.

**Figure 1:**
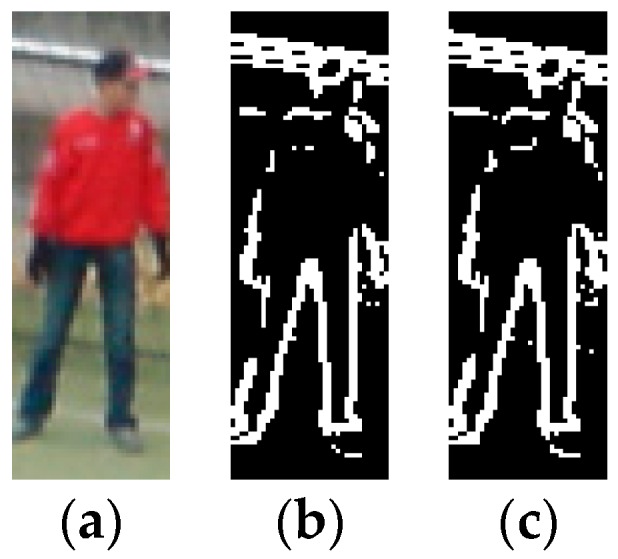
Reconstructed human image from CENTRIST. (**a**) Original image; (**b**) Contour image; (**c**) Reconstruct image.

The CENTRIST feature is robust with regard to illumination changes and gamma variations. It is a powerful tool to capture global local structures and contours beyond the small 3 × 3 range. Figure 1a,b shows a 108 × 36 human image and its contour. We divide this image into 12 × 4 blocks, so each block has 81 pixels. We can find a similar image that has the same pixel intensity histogram and CENTRIST descriptor through a reconstruction algorithm [40]. As shown in Figure 1c, the reconstructed image is similar to the original image. The global characteristics of the human contour are well preserved in spite of errors in the left part of the human. From this example, we know that CENTRIST not only encodes important information but also implicitly encodes the global contour encourages us to use it as a suitable representation for object detection. The speed issue of feature extraction is very important, because real-time detection is the prerequisite in the PPS. Comparing with SIFT and HOG, CENTRIST not only exhibits good performance, it is easy to implement and evaluates extremely quickly.

**Figure 2:**
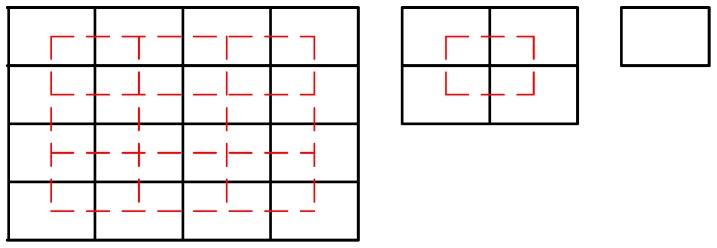
Spatial pyramid for CENTRIST.

In order to capture the rough global information of an image, CENTRIST generally uses the spatial pyramid framework, which is an extension of the SPM scheme in [41]. As shown in Figure 2, it rescales the image size for different level and the overlapped region indicated by dash lines, so it contains 31 blocks of the same size in 3 levels. CENTRISTs extracted from all the blocks are then concatenated to form the final feature vector. Features pyramid representations have proven effective for visual processing tasks such as denoising, texture analysis and recognition [42].

### 2.2. Sparse Representation Classifier

SRC is a nonparametric learning method similar to nearest neighbor (NN) and nearest subspace (NS). The basic idea is that training samples form a training matrix as a dictionary and then the testing sample can be spanned by this dictionary sparsely. In other words, a testing sample is only related to few columns in this dictionary. SRC has been successfully applied to human frontal face recognition in [36]. They experimentally show that SRC has better classification performance, which can effectively overcome the small samples and overfitting problem of NN and NS.

Assume that there are a set of training samples $\left\{(x_{i,}l_{i})|x_{i}\in {ℜ}^{m},l_{i}\in \{1,2,\cdots ,c\},i=1,2,\cdots n\right\}$, where c is the number of classes, *m* is the dimensionality of the input sample, $l_{i}$ is label corresponding to $x_{i}$. Given a test sample y, the goal is exactly to predict the label of y from the given c–class training samples. Now we arrange the *j*th class training samples as columns of a matrix $X_{j}=[x_{j,1},\cdots ,x_{j,n_{j}}]\in {ℜ}^{m\times n_{j}},j=1,2,\cdots ,c$, where $x_{j,i}$ denotes the sample belonging to the *j*th class, and $n_{j}$ is the number of the class training samples. Define a new dictionary matrix X for all training samples.

(2) $$X=[X_{1},X_{2},\cdots ,X_{c}]\in {ℜ}^{m\times n}$$

where $n=\sum_{j=1}^{c}n_{j}$. The representation model of SRC could be written as

(3) $$\hat{\alpha }=\arg\underset{\alpha }{\min}\left\{{\left\|y-X\alpha \right\|}_{2}^{2}+\lambda {\left\|\alpha \right\|}_{1}\right\}$$

where α is the vector of coefficients which is expected to be sparse, ${\left\|\cdot \right\|}_{1}$ denotes the L1-norm.

The classification of y is done by

(4) $$\mathrm{identity}(y)=\arg\underset{j}{\min}\left\{{\left\|y-X_{j}\delta_{j}(\hat{\alpha })\right\|}_{2}\right\}$$

where $\delta_{j}(\cdot ):{ℜ}^{n}\to {ℜ}^{n_{j}}$ is the characteristic function that selects from $\hat{\alpha }$ the coefficients associated with the *j*th class. When the L1-norm changes L2-norm in Equation (3), we can get the collaborative representation classifier (CRC). It is shown in [39] that CRC has comparable accuracy to SRC in face recognition without occlusion but with much faster speed. For occlusion or corruption, Robust-SRC [39] classifies the occluded image y with

(5) $$\mathrm{identity}(y)=\arg\underset{j}{\min}\left\{{\left\|y-X_{j}\delta_{j}(\hat{\alpha })-X_{e}{\hat{\alpha }}_{e}\right\|}_{2}\right\}$$

where

(6) $$[\hat{\alpha },{\hat{\alpha }}_{e}]=\arg\underset{\alpha ,\alpha_{e}}{\min}\left\{{\left\|y-X\alpha -X_{e}\alpha_{e}\right\|}_{2}^{2}+\lambda {\left\|[\alpha ,\alpha_{e}\right\|}_{1}\right\}$$

and $X_{e}$ is an occlusion dictionary to code the outliers and could set as the identity matrix.

## 3. Hierarchical Kernel Sparse Representation

### 3.1. Hierarchical Features Extraction

The appearance of pedestrians exhibits very high variability since they can change pose, wear different clothes, carry different objects, and have a considerable range of sizes. Pedestrians can be partially occluded by common urban elements, such as parked vehicles or street furniture. Classical features extraction methods such as the HOG mainly consider the global scatter of samples and may fail to reveal object local discriminative structures. In this section, we propose a very effective hierarchical features extraction (HFE) technique to capture discriminative structures at varying scales.

**Figure 3:**
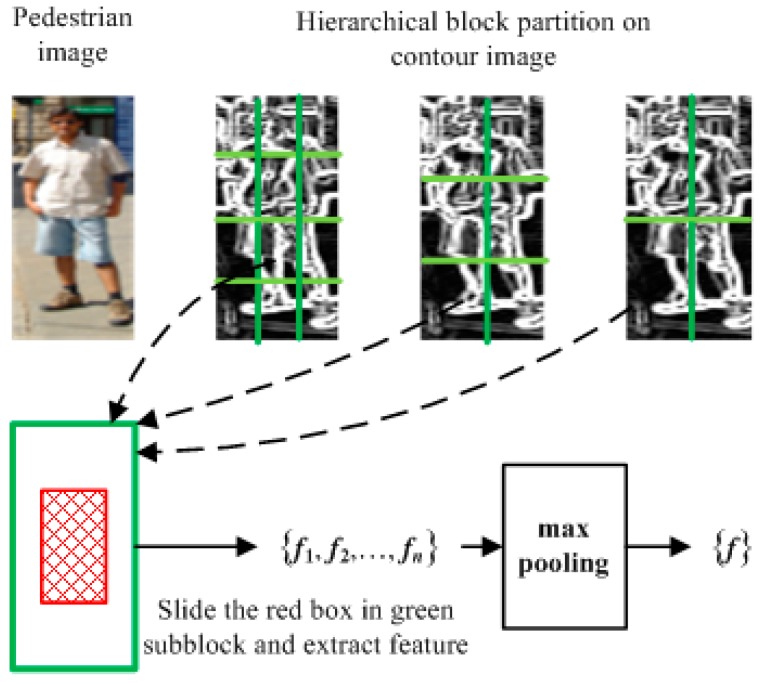
Illustration of proposed HFE.

Firstly, we adopt S + 1 level block partition, where *s* = 0, 1, …, S. That is to say, in the *s*th level, the whole image is divided into *p*_s_ × *q*_s_ blocks, each of which is further partitioned into *p*_s_ × *q*_s_ sub-blocks. Different from the partition of spatial pyramid, such as 1 × 1, 2 × 2, 4 × 4, we adopt a more flexible partition. As shown in the first row of Figure 3, for example, the partition of the sample can be made as 2 × 2, 3 × 2, and 4 × 3, respectively, with 22 blocks of three different sizes in total. This kind of partition could flexibly set the number of blocks in each scale and is expected to capture more spatial discrimination information than the spatial pyramid. As shown in the second row of Figure 3, in each sub-block we first create a sequence of 3 × 3 sliding boxes (e.g., the red box shown in Figure 3), and then compute the CENTRIST descriptor of each box’s local feature. In this paper, HFE is defined as the one with the following setting: *p*_s_ = 2 and *q*_s_ = 2 for partition scale *s* = 0 and 1; *p*_s_ = 1 and *q*_s_ = 1 for *s* > 1.

Pooling techniques are widely used in object and in image classification to extract invariant features [43,44]. In this paper, the max pooling operation is operated on a series of local features generated in each partitioned sub-block. Denoted by *f*_i_ is the feature vector extracted from the *i*th sliding box, and suppose that there are n feature vectors, *f*_1_, *f*_2_, …, *f*_n_, which are extracted from all possible sliding boxes in this sub-block, and then the final output feature vector, denoted by *f*, after max pooling is

(7) $$\left\{f\}=\max\{\{f_{1}\},\{f_{2}\},\cdots ,\{f_{n}\}\right\}$$

Let us suppose that the sample is partitioned into B blocks in total. In each block, after extracting the max pooling (MP) features of every sub-block, we concatenate the MP features of all sub-blocks as the output feature vector. Denoted by *y*_i_ is the output feature vector in the *i*th block. Then the concatenation of all feature vectors extracted from all blocks, i.e., *y* = [*y*_1_, *y*_2_, …, *y*_B_] could be taken as the descriptor of the sample image. For example, the size of original image is 128 × 48. The whole image is divided into three level as 2 × 2, 3 × 2, and 4 × 3, totally 22 blocks. Each block is partitioned into 2 × 2 sub-blocks, for a total of 88 sub-blocks. Each sub-block extracts 16 dimensions of the feature vector. Then, the final image descriptor has 1408 dimensions through concatenating all feature vectors. The proposed HFE method could not only introduce more spatial information because of its use of hierarchical structures, but also enhance the robustness with regard to varying illumination and appearance because of its use of max pooling.

### 3.2. Robust Kernel Sparse Representation

SRC behaves well in human frontal face recognition. However, SRC has poor classification ability even for the linearly separable task in which the data from different classes have the same direction. The main reason is that the data in the same direction would overlap each other after the normalization process, so we cannot essentially distinguish them. To resolve this problem occurring in SRC, the kernel trick is introduced into SRC and generates a kernel sparse representation-based classifier [45].

Only a kernel satisfying Mercer’s condition is called a Mercer kernel which is generally used in kernel methods. In other words, a Mercer kernel is continuous, symmetric, positive semi definite kernel function. Usually, a Mercer kernel function *k*(.) can be expressed as

(8) $$k(x,z)=\varphi {\left(x\right)}^{T}\varphi (z)$$

where *T* denotes the transpose of a matrix or vector, *φ* is the implicit nonlinear mapping associated with the kernel function *k*(.), which maps the feature vectors **x** and **z** to a higher dimensional feature space. The kernel function is actually Euclidian vector inner product between two image features. In kernel methods, we do not need to know what is and just adopt the kernel function Equation (8). It has been shown that histogram intersection kernel and Chi-square kernel are more powerful than other kernel function in classification [27]. Therefore, more discriminant information embedded in HFE could be exploited if the histogram intersection kernel or Chi-square kernel could be adopted in the SRC. The histogram intersection kernel $k_{HIK}$ and Chi-square kernel $k_{C}$ are defined as follows:

(9) $$k_{HIK}(x,z)=\sum_{i=1}^{n}\min(x_{i},z_{i}),\;k_{C}(x,z)=\sum_{i=1}^{n}\frac{2x_{i}z_{i}}{x_{i}+z_{i}}$$

After the HFE-based features extraction on the query image, B blocks of multiple partitions are obtained, and B sub-feature vectors, denoted by *y*_1_, *y*_2_, …, *y*_B_, are extracted. Similarly, for each of the training samples, we can extract the sub-feature vectors, and let us denote by **X***_i_* the matrix formed by all the sub-feature vectors of the *i*th block from all training samples. Taking the *i*th block as an example, the kernel representation of *y_i_* over the matrix X*_i_* could be formulated as

(10) $$\underset{\alpha }{\min}\left\{{\left\|\varphi (y_{i})-\varphi (X_{i})\alpha_{i}\right\|}_{2}^{2}+\lambda {\left\|\alpha_{i}\right\|}_{1}\right\}$$

where $\alpha_{i}$ is the coding coefficient vector in the high dimensional feature space mapped by the kernel function *φ*. Let $k_{XX}$ be a n × n matrix with ${\left\{k_{XX}\right\}}_{ij}=k(X_{i},X_{j})$ and $k_{Xy}$ be a n-dimensional vector with ${\left\{k_{Xy}\right\}}_{i}=k(X_{i},y)$. Equation (4) can be written as:

(11) $$\underset{\alpha }{\min}\left\{k(y_{i},y_{i})+{\alpha_{i}}^{T}k_{XX}\alpha_{i}-2{\alpha_{i}}^{T}k_{Xy_{i}}+\lambda {\left\|\alpha_{i}\right\|}_{1}\right\}$$

If we enforce $\alpha_{i}=\alpha_{j}$ for different blocks *i* ≠ *j*, i.e., we assume that the different blocks *y_i_* extracted from the same test sample have the same representation over their associated matrix **X***_i_*, then kernel representation of the query image by combining all the block features could be written as

(12) $$\underset{\alpha }{\min}\left\{{\left\|\varphi (y_{1})\varphi (y_{2})\cdots \varphi (y_{B})-\varphi (X_{1})\varphi (X_{2})\cdots \varphi (X_{B})\alpha \right\|}_{2}^{2}+\lambda {\left\|\alpha \right\|}_{1}\right\}$$

where **α** is the coding coefficient vector of the query sample. The above model seeks a regularized representation for a mapped feature under the mapped basis in the high dimensional space.

### 3.3. Occlusion Solution

In the kernel representation model Equation (12), the L2-norm is used to measure the representation residual. Such a kernel representation is effective when there are no outliers in the query image. However, partial occlusion or noise can often appear in the query pedestrian image. In such case, the block in which occlusion appear will have a big representation residual, reducing the role of clean blocks in the final classification. In short, the representation model in Equation (12) is very sensitive to partial occlusion.

To make the kernel representation robust to partial occlusion and noises, we propose to adopt some robust fidelity term in the modeling. Denoted by *e* = [*e*_1_, *e*_2_, …, *e*_B_] the representation residual vector, where *e_i_* is the kernel representation residual of the *i*th block:

(13) $$e_{i}={\sqrt{\left\|\varphi (y_{i})-\varphi (X_{i})\alpha_{i}\right\|}}_{2}^{2}$$

We assume that *e_i_* is independent from *e_j_* if *i ≠ j* as they represent the representation residuals of different blocks.

The proposed weighted kernel sparse representation (WKSR) can then be formulated as

(14) $$\underset{\alpha }{\min\;}\omega (e)+\lambda {\left\|\alpha \right\|}_{1}$$

where $\omega (e)=\sum_{i=1}^{B}\omega (e_{i})$ and the weight function ω(⋅) is expected to be insensitive to the outliers in the query sample. A good weight function should be robust to outliers, i.e., $\omega (e_{i})$ has a large value when | *e_i_* | is small (e.g., blocks without outliers), and a small value when | *e_i_* | is big (e.g., blocks with outliers). The widely used Gaussian function can be chosen as the weight function

(15) $$\omega (e_{i})=\frac{1}{\sqrt{2\pi \sigma^{2}}}\exp\left(-\frac{{e_{i}}^{2}}{2\sigma^{2}}\right)$$

The above weight function could effectively assign the outliers with large representation residuals low weights, and assign inliers with small representation residuals high weights (here the weight value is normalized to the range of [0, 1]). It should be noted that the weight values of each testing sample are estimated online, and there is not a training phase of them.

With the above development, Equation (12) could be rewritten as

(16) $$\underset{\alpha }{\min}\sum_{i=1}^{B}\omega_{i}\left\|\varphi (y_{i})-\varphi (X_{i})\alpha_{i}\right\|+\lambda {\left\|\alpha_{i}\right\|}_{1}$$

where $\omega_{i}$ is $\omega (e_{i})$ computed by Equation (15) with $e_{i}={\sqrt{\left\|\varphi (y_{i})-\varphi (X_{i})\alpha_{i}\right\|}}_{2}^{2}$ and $\alpha_{i}$ is an known coding coefficient vector. Here σ are scalar parameters, which could be set as a constant value or automatically updated. σ is usually set as $1/\sqrt{2\pi }$ to make the weight close to 1 when *e_i_* = 0.

With the defined kernel matrix $k_{XX}$ and kernel vector $k_{Xy}$, Equation (16) could be re-written as

(17) $$\underset{\alpha }{\min}\left\{\sum_{i=1}^{B}\omega_{i}k(y_{i},y_{i})+{\alpha_{i}}^{T}\sum_{i=1}^{B}\omega_{i}k_{X_{i}X_{i}}\alpha_{i}-2{\alpha_{i}}^{T}\sum_{i=1}^{B}\omega_{i}k_{X_{i}y_{i}}+\lambda {\left\|\alpha_{i}\right\|}_{1}\right\}$$

From Equation (17) we can see that the proposed WKSR methods could exploit the discrimination information in the mapped higher dimensional feature space; at the same time, the weight $\omega_{i}$ can effectively remove the outliers’ effect on computing the coefficient vector. 

The coefficient vector **α** is regularized by L1-norm. Efficient feature-sign search algorithm [46] could be used to solve the sparse coding problem of Equation (17). The solving of WKSR is an iterative and alternative process: the weight value is estimated via Equation (15) with known sparse coefficient, and then the sparse coefficient is computed via Equation (17) with known weight value. After getting the solution $\hat{\alpha }$ after some iteration, the classification of the query sample is done via

(18) $$\mathrm{identity}(y)=\arg\underset{j}{\min}\left\{\sum_{i=1}^{B}\omega_{i}\varepsilon_{i,j}\right\}$$

where $\varepsilon_{i,j}={\left\|\varphi (y_{i})-\varphi (X_{i,j}){\hat{\alpha }}_{j}\right\|}_{2}^{2}$ is the *i*th-block kernel representation residual associated with the *j*th class. $X_{i}=[X_{i,1},X_{i,2},\cdots ,X_{i,c}]$ with $X_{i,j}$ being the sub-matrix of $X_{i}$ associated with the *j*th class, ${\hat{\alpha }}_{j}$ being the representation coefficient vector associated with the *j*th class. From Equation (18) it can be seen that the classification criteria is based on a weight sum of kernel representation residuals, which utilizes both the discrimination power of kernel representation in high-dimensional feature space and the insensitiveness of robust representation to outliers. In addition, the kernel representation residual, $\varepsilon_{i,j}$ could be rewritten as

(19) $$\varepsilon_{i,j}=k(y_{i},y_{i})+{{\hat{\alpha }}_{j}}^{T}k_{X_{i,j}X_{i,j}}{\hat{\alpha }}_{j}-2{{\hat{\alpha }}_{j}}^{T}k_{X_{i,j}y_{i}}$$

### 3.4. Proposed Classification Algorithm

For pedestrian classification, the goal is to determine a class label for a query image. We consider a two class problem with classes C0 (pedestrian) and C1 (nonpedestrian). The whole algorithm of the proposed pedestrian classification is summarized in Algorithm 1.
**Algorithm 1:** Weighted Kernel Sparse Representation Classifier1. Hierarchical Features Extraction based on CENTRIST2. WKSR: Initialize the weight in each block as 1:
$\omega_{i}=1$
While not converge, do (a) Compute weighted kernel sparse representation ${\hat{\alpha }}_{i}=\arg\underset{\alpha }{\min}\left\{\sum_{i=1}^{B}\omega_{i}k(y_{i},y_{i})+{\alpha_{i}}^{T}\sum_{i=1}^{B}\omega_{i}k_{X_{i}X_{i}}\alpha_{i}-2{\alpha_{i}}^{T}\sum_{i=1}^{B}\omega_{i}k_{X_{i}y_{i}}+\lambda {\left\|\alpha_{i}\right\|}_{1}\right\}$ (b) Compute the reconstruction residual $e_{i}={\sqrt{\left\|\varphi (y_{i})-\varphi (X_{i})\alpha_{i}\right\|}}_{2}^{2}=k(y_{i},y_{i})+{{\hat{\alpha }}_{j}}^{T}k_{X_{i}X_{i}}{\hat{\alpha }}_{j}-2{{\hat{\alpha }}_{j}}^{T}k_{X_{i}y_{i}}$ (c) Compute the weight value $\omega (e_{i})=\frac{1}{\sqrt{2\pi \sigma^{2}}}\exp\left(-\frac{{e_{i}}^{2}}{2\sigma^{2}}\right)$ (d) Checking convergence condition $\sum_{i=1}^{B}({\omega_{i}}^{\left(t\right)}-{\omega_{i}}^{\left(t-1\right)}{)}^{2}/\sum_{i=1}^{B}{\left({\omega_{i}}^{\left(t-1\right)}\right)}^{2}<\tau$ where τ is a small positive scalar and ${\omega_{i}}^{\left(t\right)}$ is the weight value of *i*th block in the iteration t.3. **Do classification**
$\text{identity}=\arg\underset{j}{\min}\left\{\sum_{i=1}^{B}\omega_{i}k(y_{i},y_{i})+{{\hat{\alpha }}_{j}}^{T}\sum_{i=1}^{B}\omega_{i}k_{X_{i,j}X_{i,j}}{\hat{\alpha }}_{j}-2{{\hat{\alpha }}_{j}}^{T}\sum_{i=1}^{B}\omega_{i}k_{X_{i,j}y_{i}}\right\},j=0,1$ where $X_{i,j}$ the sub-matrix of $X_{i}$ associated with the *j*th class, ${\hat{\alpha }}_{j}$ being the representation coefficient vector associated with the *j*th class.


The algorithm includes three steps: (1) the first step extracts the discrimination information using the proposed HFE; (2) the second step performs WKSR; and (3) the last step performs classification. The second step is an iterative process. Through experiments, we found that this process converges fast. For instance, when there is no occlusion, only two or three iterations are needed, and when there is occlusion in the query image, approximately ten iterations can lead to a good solution.

Compared with the HOG + SVM and SRC approaches, the proposed WKSR method attenuates the problems of the query images with corrupted, occluded or largely varied appearances that may mislead the representation and classification. The running speed of HFE − WKSR is very fast. Under the programming environment of MATLAB version R2010a in a desktop of 3.07-HHz CPU with 8-GHz RAM, the running time of SRC and HFE − WKSR using feature-sign search algorithm [46] is compared in Table 1. In the experiment of INRIA database (refer to Section 4 for the detailed experimental setting), the average running time of HOG + SVM is 0.1806 s; the average running time of HFE + SRC and HFE − WKSR is 0.1239 s and 0.1372 s, respectively. In the experiment of Daimler datasets with partial occlusion (refer to Section 4 for the detailed experimental setting), the average running time of HFE + SRC and HFE − WKSR is 0.0403 s and 0.0463 s, respectively, which is much less than that of HOG + SVM (0.0682 s).

## 4. Experimental Results

In this section, we present experimental results on benchmark pedestrian databases to illustrate the effectiveness of our method. In Section 4.1, we discuss the parameter setting. In Section 4.2, we present the experimental results on INRIA databases captured in high definition digital camera. In Section 4.3, we present the experimental results on Daimler dataset captured in mobile recoding setup to demonstrate the robustness of HFE − WKSR to varied illumination, background and appearance. Then in Section 4.4, we test the robustness of HFE − WKSR against partial occlusion in INRIA random block occlusion and Daimler Occlusion datasets.

### 4.1. Parameter Setting

The proposed method consists of two main procedures: hierarchical feature extraction (HFE) and WKSR. With no specific instruction, the parameters of HFE–WKSR are set as shown in Table 2. In feature extraction, the histogram of CENTRIST encoded on the raw image is used as the local features, and the number of histogram bins for each sub-block is set to 16. In the proposed hierarchical features extraction method, we set *s* = 0, *p*_0_ = 4, and *q*_0_ = 4 for INRIA and Daimler dataset with non-occlusion images. For Daimler and INRIA dataset with partial occlusion images, we set *s* = 2, and (*p*_s_, *q*_s_) ={(4, 4, (3, 2), (2, 2)} for *s* = {0, 1, 2}. In the procedure of WKSR, the histogram intersection kernel [42] is used as the kernel function. In the Gaussian weight, we set σ=0.5 for samples with occlusion and σ=0.4 for samples without occlusion. The convergence parameter τ and the Lagrange multiplier λ is empirically set as 0.7 and 0.005, respectively. The other parameters are obtained by cross-validation. We use randomly selected 100 of all labeled samples as the training set and 500 samples as test set, then vary level from 1 to 4, bin number form 8, 16 and 32, weight from 0.2 to 0.8. Each experiment is repeated five times using different random sampling. Finally, we determine parameters setting according to time consumption and classification accuracy.

### 4.2. Pedestrian Classification on INRIA Dataset

**Figure 4:**
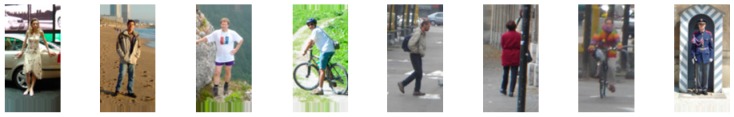
Some samples of INRIA dataset.

We first evaluate the performance of the proposed algorithm on INRIA databases captured in static digital camera, which has been widely used for pedestrian/human detection evaluation in recent years. The original SRC and SVM with HOG feature [7] is used as the baseline methods, and we then apply the proposed HFE feature to SRC [36], CRC [39], histogram intersection kernel-based support vector machine (HIKSVM) as its similarity measurement, and compare them with the proposed HFE − WKSR. INRIA consists of 1758 positives and 1685 negatives images captured under various view and illumination conditions. Example of images from the dataset are shown in Figure 4. In our experiment, *N* samples are randomly chosen as training samples and 500 of the remaining images are randomly chosen as the testing data. Here the images are normalized to 128 × 64 and the experiment for each *N* samples runs ten times.

The pedestrian classification results and mean recognition accuracy of all the competing methods are listed in Table 3. The proposed HFE − WKSR achieves the best performance, with more than a 4% improvement over all the others when *N* is small (e.g., 20 and 50). When 100 training samples are selected, an accuracy of 97.5% is achieved by HFE − WKSR. It could also be seen that those methods based on sparse representation (e.g., HFE − WKSR, HFE + CRC, HFE + SRC, and HOG + SRC) are more powerful than SVM-based methods.

### 4.3. Pedestrian Classification on Daimler Dataset

**Figure 5:**
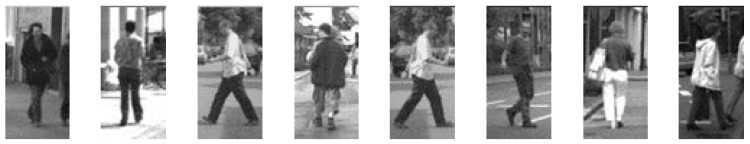
Some samples of Daimler dataset.

In this section, we test the robustness of the proposed method to real traffic scenes on Daimler databases with complex background, varied illumination and appearances. Daimler databases consists of 15,659 pedestrian and 6740 nonpedestrian samples captured from vehicle-mounted camera in an urban environment. As opposed to the INRIA dataset, nonpedestrian samples were selected by a preprocessing step from the negative samples, which match a pedestrian shape template based on the average Chamfer distance score. Both samples were scaled into a fixed size of 96 × 48 windows, and pedestrian samples include a margin of 2 pixels around. The small size of the windows, combined with motion background, makes detection on the Daimler dataset extremely challenging. Examples of images from the dataset are shown in Figure 5. In the experiment, all pedestrian samples are divided into three groups, including illumination, background and appearance change. 1000 samples are randomly chosen as training samples and 9000 of the remaining images are randomly chosen as the testing data. Here the images are normalized to 96 × 48 and the experiment for each group runs ten times.

Table 4 lists the results of all the competing methods. It can be seen that the proposed HFE − WKSR achieves the highest recognition rates, with at least 3% improvements than all the other methods, respectively. The original SRC with HOG gets the worst recognition rates, much lower than HFE + SRC. This validates that HFE is robust to misalignment to some extent. Sparse representations (e.g., CRC and SRC) combined with HFE could have approximately 10% improvements over other kinds of classifiers (e.g., HISVM, SVM). To show the effectiveness of MP, we also give the recognition rate of SLF-RKR without the step of MP in Table 4. One can see that even without MP, HFE − WKSR still outperforms HFE + SRC by 1.9% in average, whereas HFE − WKSR outperforms HFE + CRC by 2.6%. It can also be observed that the improvement introduced by MP is over 5% in each session, which clearly shows the effectiveness of the proposed MP in dealing with varied illumination, background and appearance.

### 4.4. Pedestrian Classification on Partial Occlusion Datasets

Partial occlusion is a very challenging issue in a pedestrian detection system when the subject is covered by other objects such as trees, cars and other human. One interesting property of SRC [36] is its robustness to occlusions. In this section, we test the performance of HFE − WKSR to various occlusions, including random block occlusion and real occlusion. In HFE − WKSR, the robustness to occlusion mainly comes from its iterative reweighed kernel robust representation. In this section, the weight W in each block is automatically updated.

**Figure 6:**
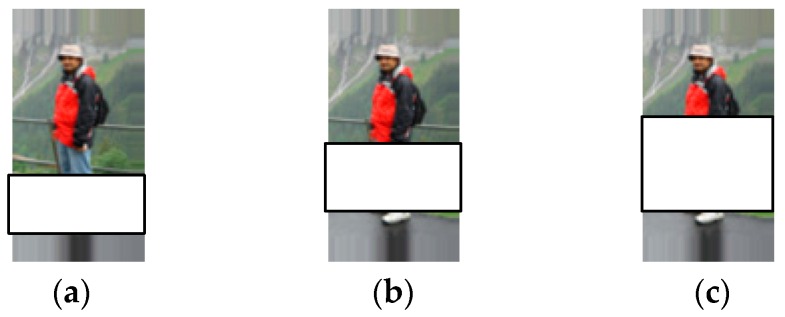
Examples of pedestrian images with random block occlusion. (**a**) 20% block occlusion; (**b**) 30% block occlusion; (**c**) 40% block occlusion.

(1) Pedestrian classification with random block occlusion. In the database of INRIA, we chose 100 non-occlusion images with normal-to-moderate lighting conditions for training, and 500 of the remaining images are randomly chosen for testing. Similar to the settings in [36], we simulate various levels of contiguous occlusion, from 0% to 50%, by replacing a randomly located square block of each testing image with an unrelated image, as illustrated in Figure 6, where (a) shows a pedestrian image with 20% block occlusion, (b) shows a pedestrian image with 30% block occlusion and (c) shows a pedestrian image with 40% block occlusion. Here the location of occlusion is randomly chosen for each image and is unknown to each algorithm, and the image size is normalized to 128 × 64.

Table 5 lists the classification results versus various levels of occlusions. Here λ of HFE − WKSR is set as 0.1. From Table 5, we can see that almost all methods could correctly classify most of the testing samples when occlusion level is from 10% to 20%. However, when occlusion percentage is larger than 20%, the advantage of HFE − WKSR over other methods becomes significant. For instance, when occlusion is 40%, HFE − WKSR could achieve at least 84% recognition accuracy, compared with at most 72.5% for other methods. For HFE − WKSR, when there is 50% block occlusion, it can still achieve a recognition rate of over 75%. This clearly demonstrates the effectiveness of the proposed HFE − WKSR method to deal with partial occlusion.

**Figure 7:**
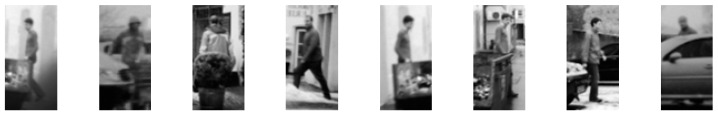
Examples of pedestrian images with real occlusion in Daimler partially occluded set.

**Figure 8:**
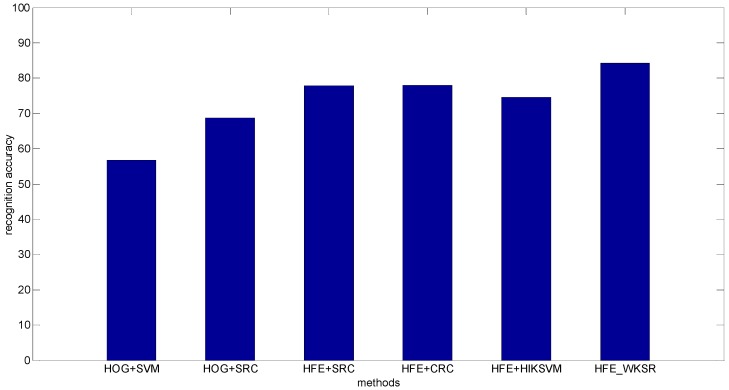
Classification Results on Daimler partially occluded set.

(2) Pedestrian classification real occlusion: The Daimler dataset is divided into partially occluded set and non-occluded test set. The partially occluded test set contains 11,160 pedestrians and 16,253 non-pedestrians. Example of images from the dataset are shown in Figure 7. Figure 8 shows the classification results. It can be seen that the proposed methods achieve 84.2% recognition accuracy, much higher than the state-of-the-art results, for example, 56.8% (HOG + SVM) and 68.7% (HOG + SRC), and 77.8% (HFE + SRC) and 78.0% (HFE + CRC), and 74.6%(HFE + HIKSVM). The improvement of HFE − WKSR over all the other methods is at least 6%, which clearly shows the superior classification ability of HFE − WKSR.

## 5. Conclusions

Because a vision-based pedestrian protection system (PPS) is low in cost, and is not influenced by temperature, it has extensive applications in autonomous vehicles. Pedestrian classification is a key technology for PPS. In this paper, we proposed a novel HFE − WKSR model for pedestrian classification. A robust representation model for image outliers (e.g., occlusion and noise) was built in the kernel space, and a hierarchical features extraction based on the CENTRIST descriptor was proposed to capture the discriminative structures of object. A max pooling operation is used to enhance the invariance of the local pattern feature to varying illumination and appearance. We evaluated the proposed method in different conditions, including variations of illumination, view, appearance, as well as block occlusion. One big advantage of the proposed method is its high recognition rates and robustness against various occlusions. The extensive experimental results demonstrated that HFE − WKSR is superior to state-of-the-art methods and has great potential to be applied in practical pedestrian protection systems.

## Figures and Tables

**Table 1:** Average running time (s).

Method	INRIA	Daimler with Occlusion
HOG + SVM	0.1806	0.0682
HFE + SRC	0.1239	0.0403
HFE − WKSR	0.1372	0.0463

**Table 2:** Parameters of HFE − WKSR.

Procedure		Parameters
Feature extraction	Hierarchical partition	P_0_ = 4, Q_0_ = 4 when S = 0 P_0_ = 4, Q_0_ = 4; P_1_ = 3, Q_1_ = 2; P_2_ = 2, Q_2_ = 2 when S = 2
	Histogram bin number	16
WKSR	Kernel function	Histogram intersection kernel
	Weight	σ=0.4 for non-occlusion σ=0.5 for occlusion
	convergence	τ=0.7
	Lagrange multiplier	λ=0.005

**Table 3:** Classification results for INRIA database.

N	20	50	100
HOG + SVM	45.2	53.6	62.5
HOG + SRC	72.8	77.1	82.9
HFE + SRC	84.2	88.9	91.3
HFE + CRC	85.3	87.9	90.8
HFE + HIKSVM	62.7	68.2	77.9
HFE − WKSR	90.3	94.4	97.5

**Table 4:** Classification Results on Daimler database.

Group	Illumination	Background	Appearance
HOG + SVM	58.7	55.2	46.3
HOG + SRC	75.4	86.6	73.5
HFE + SRC	84.5	86.4	83.2
HFE + CRC	85.4	85.5	81.2
HFE + HIKSVM	73.5	76.3	68.3
HFE − WKSR	94.6	92.5	90.3
HFE − WKSR (without MP)	88.3	87.1	84.5

**Table 5:** Classification results on block occlusion.

Occlusion	10%	20%	30%	40%	50%
HOG + SVM	57.2	53.6	42.9	38.3	32.4
HOG + SRC	72.3	68.2	55.4	48.2	47.9
HFE + SRC	83.2	80.8	76.3	72.5	68.1
HFE + CRC	81.3	76.5	73.2	71.6	67.2
HFE + HIKSVM	75.2	71.3	68.2	63.3	61.4
HFE − WKSR	93.2	91.5	88.2	82.3	75.4

## References

[1] David G., Antonio M.L., Angel D.S. (2010). Survey of Pedestrian Detection for Advanced Driver Assistance Systems. IEEE Trans. Pattern Anal. Mach. Intell..

[2] Dollar P., Wojek C., Schiele B., Perona P. (2012). Pedestrian Detection: An Evaluation of the State of the Art. IEEE Trans. Pattern Anal. Mach. Intell..

[3] Miron A., Rogozan A., Ainouz S., Bensrhair A., Broggi A. (2015). An Evaluation of the Pedestrian Classification in a Multi-Domain Multi-Modality Setup. Sensors.

[4] Ess A., Leibe B., Gool L.V. Depth and Appearance for Mobile Scene Analysis. Proceedings of the 2007 IEEE 11th International Conference on Computer Vision.

[5] Wojek C., Walk S., Schiele B. Multi-Cue Onboard Pedestrian Detection. Proceedings of the IEEE Conference on Computer Vision and Pattern Recognition.

[6] Enzweiler M., Gavrila D.M. (2009). Monocular Pedestrian Detection: Survey and Experiments. IEEE Trans. Pattern Anal. Mach. Intell..

[7] Dalal N., Triggs B. Histograms of Oriented Gradients for Human Detection. Proceedings of the IEEE Conference on Computer Vision and Pattern Recognition.

[8] Lee Y.S., Chan Y.M., Fu L.C., Hsiao P.Y. (2015). Near-Infrared-Based nighttime pedestrian detection using grouped part models. IEEE Trans. Intell. Trans. Syst..

[9] Hurbey P., Waldron P., Morgan F., Jones E., Glavin M. (2015). Review of pedestrian detection techniques in automotive far-infrared video. IET Intell. Trans. Syst..

[10] Etinger A., Balal N., Litvak B., Einat M., Kapilevich B., Pinhasi Y. (2014). Non-Imaging MM-Wave FMCW Sensor for Pedestrian Detection. IEEE Sens. J..

[11] Kim B., Choi B., Park S., Kim H. (2016). Pedestrian/Vehicle Detection Using a 2.5-D Multi-Layer Laser Scanner. IEEE Sens. J..

[12] Gandhi T., Trivedi M.M (2007). Pedestrian Protection Systems: Issues, Surveys and Challenges. IEEE Trans. Intell. Trans. Syst..

[13] Marr D. (1982). Vision: A Computational Investigation into the Human Representation and Processing of Visual Information.

[14] Viola P., Jones M., Snow D. (2005). Detecting pedestrians using patterns of motion and appearance. Int. J. Comput. Vis..

[15] Serre T., Wolf L., Bileschi S., Riesenhuber M., Poggio T. (2007). Object recognition with cortex-like mechanisms. IEEE Trans. Pattern Anal. Mach. Intell..

[16] Tuzel O., Porikli F., Meer P. (2008). Pedestrian detection via classification on Riemannian manifolds. IEEE Trans. Pattern Anal. Mach. Intell..

[17] Wang X., Han T.X., Yan S. An HOG-LBP human detector with partial occlusion handling. Proceedings of the IEEE 12th International Conference on Computer Vision.

[18] Wu B., Nevatia R. Detection of multiple, partially occluded humans in a single image by Bayesian combination of edgelet part detectors. Proceedings of the IEEE International Conference on Computer Vision.

[19] Sabzmeydani P., Mori G. Detecting pedestrians by learning shapelet features. Proceedings of the IEEE Conference on Computer Vision and Pattern Recognition.

[20] Wu J.X., Liu N., Geyer C., Rehg J.M. (2013). C4: A Real-time Object Detection Framework. IEEE Trans. Image Proc..

[21] Ye Q., Jiao J., Zhang B. Fast Pedestrian detection with multi-scale orientation features and two-stage classifiers. Proceedings of the IEEE International Conference on Image Processing.

[22] Wojek C., Schiele B. A Performance Evaluation of Single and Multi-Feature People Detection. Proceedings of the 30th DAGM Symposium Munich.

[23] Walk S., Majer N., Schindler K., Schiele B. New Features and Insights for Pedestrian Detection. Proceedings of the IEEE Conference on Computer Vision and Pattern Recognition.

[24] Wu B., Nevatia R. Optimizing Discrimination-Efficiency Tradeoff in Integrating Heterogeneous Local Features for Object Detection. Proceedings of the IEEE Conference on Computer Vision and Pattern Recognition.

[25] Dollar P., Tu Z., Perona P., Belongie S. Integral Channel Features. Proceedings of the British Machine Vision Conference.

[26] Enzweiler M., Eigenstetter A., Schiele B., Gavrila D.M. Multi-cue pedestrian classification with partial occlusion handling. Proceedings of the IEEE Conference on Computer Vision and Pattern Recognition.

[27] Maji S., Berg A., Malik J. (2013). Efficient classification for Additive Kernel SVMs. IEEE Trans. Pattern Anal. Mach. Intell..

[28] Gavrila D.M., Munder S. (2007). Multi-cue pedestrian detection and tracking from a moving vehicle. Int. J. Comput. Vis..

[29] Mikolajczyk K., Schmid C., Zisserman A. Human detection based on a probabilistic assembly of robust part detectors. Proceedings of the 8th European Conference on Computer Vision.

[30] Munder S., Gavrila D.M. (2006). An experimental study on pedestrian classification. IEEE Trans. Pattern Anal. Mach. Intell..

[31] Xu Y.W., Cao X.B., Qiao H. (2011). An efficient tree classifier ensemble-based approach for pedestrian detection. IEEE Trans. Syst. Man Cybern. Part B: Cybern..

[32] Felzenszwalb P., Girshick R., McAllester D., Ramanan D. (2010). Object Detection with Discriminatively Trained Part Based Models. IEEE Trans. Pattern Anal. Mach. Intell..

[33] Enzweiler M., Gavrila D.M. (2011). A multilevel mixture-of-experts framework for pedestrian classification. IEEE Trans. Image Proc..

[34] Aly S., Hassan L., Sagheer A., Murase H. Partially Occluded Pedestrian Classification using Part-Based Classifiers and Restricted Boltzmann Machine Model. Proceedings of the 16th IEEE Conference on Intelligent Transportation Systems.

[35] Benenson R., Omran M., Hosang J., Schiele B. Ten years of pedestrian detection, what have we learned?. Proceedings of the 13th European Conference on Computer Vision, ECCV 2014.

[36] Wright J., Yang A., Ganesh A., Sastry S., Ma Y. (2009). Robust face recognition via sparse representation. IEEE Trans. Pattern Anal. Mach. Intell..

[37] Timo A., Abdenour H., Matti P. Face recognition with local binary patterns. Proceedings of the European Conference on Computer Vision.

[38] Liu Y.G., Ge S.Z., Li C.G., You Z.S. (2011). K-NS: A classifier by the distance to the nearest subspace. IEEE Trans. Neural Netw..

[39] Zhang L., Yang M., Feng X.C. Sparse representation or collaborative representation which helps face recognition?. Proceedings of the International Conference on Computer Vision.

[40] Wu J., Rehg J.M. (2011). CENTRIST: A visual descriptor for scene categorization. IEEE Trans. Pattern Anal. Mach. Intell..

[41] Lazebnik S., Schmid C., Ponce J. Beyond bags of features: Spatial pyramid matching for recognizing natural scene categories. Proceedings of the IEEE Conference on Computer Vision and Pattern Recognition.

[42] Piotr D., Ron A., Serge B., Perona P. (2014). Fast Feature Pyramids for Object Detection. IEEE Trans. Pattern Anal. Mach. Intell..

[43] Yang J.C., Yu K., Gong Y., Huang T. Linear spatial pyramid matching using sparse coding for image classification. Proceedings of the IEEE Conference on Computer Vision and Pattern Recognition.

[44] Han H., Han Q., Li X. (2013). Hierarchical spatial pyramid max pooling based on SIFT features and sparse coding for image classification. Int. J. Comput. Vis..

[45] Zhang L., Zhou W.D., Chang P.C., Liu J., Yan Z., Wang T., Li F.Z. (2012). Kernel sparse representation-based classifier. IEEE Trans. Signal Process..

[46] Lee H., Battle A., Raina R., Ng A.Y. Efficient sparse coding algorithm. Proceedings of the 20th Annual Conference on Neural Information Processing Systems (NIPS).

